# Meta-analysis of RNA-Seq datasets allows a better understanding of *P. tricornutum* cellular biology, a requirement to improve the production of Biologics

**DOI:** 10.1038/s41598-025-87620-5

**Published:** 2025-01-29

**Authors:** Isabelle Boulogne, Charlotte Toustou, Muriel Bardor

**Affiliations:** 1 University of Rouen Normandie, UNIROUEN, UFR des Sciences et Techniques, GlycoMEV UR4358, Innovation Chimie Carnot, Fédération de Recherche Normandie-Végétal FED 4277, 76821 Mont-Saint-Aignan, France; 2 Alga Biologics, CURIB, 25 Rue Tesnières, 76821 Mont Saint Aignan Cedex, France; 3 Present Address: ECOTERCA - ÉCOlogie TERrestre CAribéenne, Université des Antilles, Faculté des Sciences Exactes et Naturelles, Campus de Fouillole, BP 592, 97159 Pointe-à-Pitre Cedex, France

**Keywords:** Meta-analysis, *P. tricornutum*, Bioproduction, Biologics, Glycosylation, Glycobiology, Glycobiology, RNA, Marine biology, Bioinformatics

## Abstract

The marine diatom *Phaeodactylum tricornutum* is currently used for various industrial applications, including the pharmaceutical industry as a cost-effective cell biofactory to produce Biologics. Recent studies demonstrated that *P. tricornutum* can produce functional monoclonal antibodies, such application is currently limited by the production yield that hinders industrialization. Therefore, it is necessary to understand and control the cell biology of *P. tricornutum* to improve the Biologics production yield. Transcriptomic analyses have recently been used by the pharmaceutical industry to improve the production of Biologics in mammalian cells, especially Biologics titer and cell productivity. Hence, in the present work, we performed a meta-analysis of seven publicly available RNA-Seq datasets from different strains of *P. tricornutum,* for which the culture conditions were chosen as similar as possible. We analyzed the differential expression of genes that are involved in biological processes that are well known to potentially impact the bioproduction and critical quality attributes of Biologics. Therefore, the expression of genes involved in the *N*-glycan biosynthesis, protein export and secretion, protein quality control and proteasome, as well as those encoding proteases were analyzed and compared. The results pave the way towards optimizing Biologics production in *P. tricornutum* and highlight that the Pt4, Pt3 Ov and Pt8 strains seem to be the most promising P. tricornutum strains.

## Introduction

The marine diatom *Phaeodactylum tricornutum* is currently used for various industrial applications such as food, nutraceuticals, cosmetics, biotechnology and bioproduction. In this context, the capacity of *P. tricornutum* to be a cost-effective cell biofactory for the production of high value-added molecules has been highlighted^1^. For example, the development of genome-editing tools in *P. tricornutum* enabled the production of bioplastics^2^ and terpenoids^3,4^*.* Furthermore, *P. tricornutum* has also been shown to be a reliable and more sustainable means of producing Biologics. Biologics are used to treat life-threatening diseases, and include gene or cell therapies as well as recombinant proteins. Due to the complexity of these recombinant proteins, their production relies on the use of living systems such as mammalian cell systems. However, alternatives such as *P. tricornutum* are currently being explored. For example, the production and secretion of monoclonal antibodies (mAbs) directed against two deadly viruses (Hepatitis B and Marburg) have been demonstrated^5–7^. More recently, the successful production of the RBD (receptor-binding domain) of the SARS-CoV-2 in *P. tricornutum* allows this microalgae to be considered as a viable platform for the production of sensitive diagnostic tools^8^. In addition, most recombinant proteins of interest to human health are glycosylated. Unlike *Chlamydomonas reinhardtii*, another microalgae used as a cell biofactory, for which the* N*-glycosylation of the recombinant erythropoietin has been shown to be very different from the human N-glycosylation^9^, *P. tricornutum* is able to synthesize *N*-glycan structures that are similar to the human ones^10,11^. Currently, the development of the production of these high value-added molecules at an industrial scale remains restrained by their limited production yield. However, since the approval of the first recombinant protein produced in Chinese Hamster Ovary (CHO) cells, these cells have benefited from more than four decades of active international research^12^. This includes CHO cell engineering, vector design and control of culture media^13–16^. Moreover, although numerous cell lines of CHO cells have been used as models since the 1950s, the genomes of CHO cell lines have only been available 13 years ago. The sequencing of the CHO-K1 cell line genome and its publication in 2011^17^ has allowed an increased use of “omics” techniques and more accurate annotation of CHO omics data^18,19^. The first applications of omics tools to CHO cell lines were mainly focused on a fundamental understanding of the cell biology. For example, initial transcriptomic works were focused on understanding the regulation of growth-phase-dependent genes, central sugar metabolism, and the *N*-glycosylation pathway^18–20^. Transcriptomic analyses are now being used directly for industrial purposes to identify genes involved in cellular pathways that could be used to optimize mAb titer and quality as well as cell productivity^21–23^.

The first fully assembled genome of *P. tricornutum* (Pt1.8.6 strain) is available since 2008^24^, allowing the generation of numerous genomic^25,26^, transcriptomic^27–31^ and even proteomic data^30,32–35^. Since 2020, genomes of several other ecotypes of *P. tricornutum* have been sequenced^36,37^. Most of the *P. tricornutum*’s omics data focus on the effect of environmental conditions, such as nutrient element depletion/starvation (nitrogen, phosphorus) or light/dark stress on gene expression for a single strain^27,30,33,35^. Most of these data are now available in the Diatomics database (https://www.diatomicsbase.bio.ens.psl.eu/) and the PhaeoEpiView database (https://phaeoepiview.univ-nantes.fr/). Unlike other diatoms, *P. tricornutum* has the unique ability to be pleiomorphic, as evidenced by the three main morphotypes observed for a single strain such as Pt3 (fusiform, oval, or triradiate)^29,38,39^. The fusiform morphotype is the most common in the natural environment. Indeed, out of the ten most studied ecotypes, seven of them are predominantly fusiform (> 95% of cells). However, some ecotypes present a natural predominance for other morphotypes, such as Pt 8, which was originally described as mainly triradiate^38^. However, this morphotype was reported to be unstable, and was not always observed in this ecotype^31,37^. Additionally, depending on the culture conditions and often under stress, other predominances are reported^38,39^. For example, the Pt 3 strain, which is derived from Pt 2, was selected for its ability to grow in freshwater media under an oval morphotype. Thus, the appearance of the oval morphotype demonstrated the ability of *P. tricornutum* to adapt to stress conditions (salinity, temperature, light). Such ability varies from one ecotype to another^38,39^. Transcriptomic and proteomic analyses of the three morphotypes of the Pt3 strain highlighted that the oval morphotype is able to secrete proteins faster, and in larger amounts as compared to the other two morphotypes^29,32^. Such observation was confirmed by microscopic analyses and protein quantification^40,41^. In addition, a comparative proteomic analysis of the proteins secreted within the culture medium, called secretome, of the three morphotypes, revealed that more proteins secreted in the culture media of the Pt 3 oval morphotype were involved in proteolysis^32^. Besides the phenotypic and genotypic differences, variations in biological processes have also been noticed between the ecotypes, such as a higher capacity of Pt4 to assimilate nitrate than the reference strain Pt 1^42^, probably due to a higher copy number of the gene encoding for the nitrate reductase^36^.

In this context, we performed a meta-analysis of transcriptomic data retrieved from the literature. Among the dozens of articles related to RNA-Seq analyses, those concerning different strains of *P. tricornutum*, for which the culture conditions were chosen as similar as possible were selected (Table 1). We especially focused on biological processes that are well-known to significantly impact the bioproduction and the critical quality attributes of Biologics. Thus, in agreement, the expression of genes involved in the *N*-glycan biosynthesis, protein export and secretion, protein quality control and proteasome as well as those encoding proteases were analyzed and compared. The results provide first elements to consider when selecting an optimal *P. tricornutum* strain for the production of Biologics.

**Table 1:** Based on our search criteria (culture conditions, ecotypes, and morphotypes), the seven RNA-seq datasets included in our study are described as follows.

Ecotype accession	Major morphotype	Medium	T°C	Photoperiod (L:D)	Light intensity (μmol m^−2^ s^−1^)	Days of culture/harvest	SRA	Reference
Pt 1.8.6	Natural fusiform 95–100%	100% artificial seawater + f/2-Si	20	12:12	100	8 days, exponentially growing cells	SRX957920 SRX957921	^27^
Pt 3 Fu	Enriched fusiform 89–91.2%	100% artificial seawater + Conway + Si	19	16:8	68	8 days, exponentially growing cells	ERR3285011 ERR3285012 ERR3285013 ERR3285014	^29^
Pt 3 Ov	Enriched oval 97.6–98.4%	10% artificial seawater + Conway + Si	19	16:8	68	8 days, exponentially growing cells	ERR3285015 ERR3285016 ERR3285017 ERR3285018	^29^
Pt 3 Tr	Enriched triradiate 76.5–77.5%	100% artificial seawater + Conway + Si	19	16:8	68	8 days, exponentially growing cells	ERR3285019 ERR3285020 ERR3285021 ERR3285022	^29^
Pt 3	Natural oval 60–75%	No details available in the paper*	20	12:12	75	Exponentially growing cells	SRR9945641	^36^
Pt 4	Natural fusiform 95–100%	No details available in the paper*	18	16:8	40	6 days (4 days in light + 2 days in darkness)	SRR5298736 SRR5298737 SRR5298738	^28^
Pt 8	Natural triradiate 80–85%	100% natural seawater + f/2	18	12:12	175	Cultures to reach 2.10^6^ cells.mL^−1^ + 2 days	SRR10120340 SRR10120339	^31^

*As not specific details were provided in the publication regarding the composition of the culture media, we assumed that the culture media were either Conway or f/2, that are medium commonly used for the culture of *P. tricornutum*. *All data were downloaded from SRA-NCBI in February 2023.*

## Results and discussion

Based on our search criteria, which included information on culture conditions, ecotypes, and morphotypes, seven publicly available RNA-Seq datasets were selected from the literature and used in the present work (Table 1). Unfortunately, we were unable to find RNA-seq data with culture conditions as similar as possible for the 10 *P. tricornutum* ecotypes (Pt1 to Pt10). The selected datasets include the RNA-Seq dataset of the strain Pt1.8.6, for which the genome has been sequenced and annotated. As this is the most widely used and studied strain, we selected it as the reference strain^24,36^. This reference strain was reported to be 95–100% fusiform. Other datasets concerning the Pt3, Pt4 and Pt8 strains were also included as they were cultured under similar conditions. Pt4 was described as mainly fusiform (95–100%) as reported for Pt1 whereas Pt3 and Pt8 were predominantly oval (60–75%) and triradiate (80–85%) morphotypes, respectively. Another RNA-seq study regarding the Pt3 strain, which was specifically enriched in the different morphotypes (Pt3 Fus, Pt3 Ov, Pt3 Tr), was also included in the study for comparison. For all the strains, RNA extractions were performed on 6 to 8 days of culture under similar conditions, using seawater supplemented with either Conway or f/2, which are very close in terms of composition. The temperature used for the cultivation was comprised between 18 and 20°C.

### Differentially expressed genes

The differential expression of genes (DEG) analysis performed in the different ecotypes of *P. tricornutum* using Pt1.8.6 as a reference (Supplemental data S1) showed that 2,284, 2,817, 1,975, 2,415, 2,332 and 2,457 genes are significantly more expressed in Pt3 Fu, Pt3 Ov, Pt3 Tr, Pt3, Pt4 and Pt8, respectively, than in the reference strain. Regarding down-regulated genes, 2,071, 2,219, 1,756, 2,460, 2,935 and 2,645 genes are significantly less expressed in Pt3 Fu, Pt3 Ov, Pt3 Tr, Pt3, Pt4 and Pt8, respectively, compared to Pt1.8.6. Overall, the number of DEG is relatively well balanced between the ecotypes and more or less expressed genes (over 2,400 DEG), except for Pt3 Tr, which presents less than 2,000 more or less expressed DEG. It should also be noted that Pt3 Ov is the ecotype with the most genes more expressed than the reference strain (2,817) and Pt4 is the ecotype with the most genes less expressed than the reference strain (2,935). The ecotypes Pt3, Pt4 and especially Pt8 also present a greater number of genes with a significantly higher difference in expression (high −log10 (*p* value)) compared to Pt3 Fu, Pt3 Ov and Pt3 Tr, especially for less expressed genes.

In order to investigate more precisely the transcriptomic differences between the ecotypes, a pathway analysis was carried out using ShinyGO (Table 2). This analysis showed that no significant enrichment was found for Pt3 Tr, either for more or less expressed genes, despite the morphotype difference between the two ecotypes (fusiform for Pt1.8.6 and triradiate for Pt3 Tr). The lack of significant enrichment can be attributed to the low number of DEGs between the two accessions. This result is in agreement with a previous transcriptomic study carried out on the 3 morphotypes of the Pt3 strain^29^. It was shown that less than 1% of the transcriptome was differentially expressed between the two morphotypes of the same ecotype. No significant enrichment was also found for Pt3 Fu and Pt3 Ov in terms of genes more expressed than the reference strain. On the other hand, cultures enriched with these morphotypes showed an enrichment in ‘Ribosome biogenesis in Eukaryotes’ and ‘Ribosome’ pathways with less expressed genes than the reference. This is also the case for Pt4 and Pt8. It should be noted that such enrichment is independent of the morphotypes, as Pt3 Ov is mainly oval, Pt4 mainly fusiform, while Pt8 is mainly triradiate. The enrichment of these pathways ‘Ribosome biogenesis in Eukaryotes’ and ‘Ribosome’ has been observed previously, but as over-expressed genes in studies dealing with the effect of naphthenic acids as a stress condition on *P. tricornutum*^43^, H_2_O_2_ on *Chlorella pyrenoidosa*, tetracycline on *Scenedesmus obliquus*^44^ or cold stress on *Neoporphyra haitanensis*^45^. The authors of these studies correlated the overexpression of these pathways with the adaptation of the microalgae to stress conditions. Pt3 and Pt8 were significantly enriched with more expressed genes than the reference strain in ‘Biosynthesis of secondary metabolites’, ‘Fatty acid biosynthesis’, ‘Porphyrin and chlorophyll metabolism’ and ‘Protein processing in endoplasmic reticulum’. On the other hand, Pt3 exhibited significant enrichment with less expressed genes than the reference strain in ‘Valine, leucine and isoleucine degradation’. Pt4 showed significant enrichments with more expressed genes than the reference strain in two pathways related to the protein life cycle: ‘Protein export’ and ‘Protein processing in endoplasmic reticulum’. In contrast, the less expressed genes of this ecotype compared to the reference strain presented enrichments in several pathways of the carbohydrate metabolism category such as ‘Citrate cycle (TCA cycle)’, ‘Pyruvate metabolism’, ‘Glycolysis / Gluconeogenesis’ and ‘Carbon metabolism’.

**Table 2:** Fold enrichment pathways analysis with DEG found in Pt3 Fu, Pt3 Ov, Pt3 Tr, Pt3, Pt4 or Pt8 (compared to Pt1.8.6) with ShinyGO 0.77 (Pathway database: KEGG, FDR cutoff 0.05).

	Pathways significantly enriched with more expressed than the reference strain genes	Pathways significantly enriched with less expressed than the reference strain genes
Pt 3 Fu	No significant enrichment found	Ribosome biogenesis in Eukaryotes Ribosome
Pt 3 Ov	No significant enrichment found	Ribosome biogenesis in Eukaryotes Ribosome
Pt 3 Tr	No significant enrichment found	No significant enrichment found
Pt 3	Fatty acid biosynthesis Ribosome Porphyrin and chlorophyll metabolism Pyrimidine metabolism Biosynthesis of cofactors Protein processing in endoplasmic reticulum Biosynthesis of secondary metabolites Metabolic pathways	Valine, leucine and isoleucine degradation
Pt 4	Protein export Protein processing in endoplasmic reticulum	Citrate cycle (TCA cycle) Pyruvate metabolism Porphyrin and chlorophyll metabolism Glycolysis / Gluconeogenesis Carbon metabolism Biosynthesis of amino acids Ribosome biogenesis in eukaryotes Ribosome Biosynthesis of secondary metabolites Biosynthesis of cofactors Metabolic pathways
Pt 8	Proteasome Photosynthesis Fatty acid biosynthesis Porphyrin and chlorophyll metabolism Protein processing in endoplasmic reticulum Biosynthesis of secondary metabolites Metabolic pathways	Ribosome biogenesis in eukaryotes

This first analysis could imply that Pt3 Fu, Pt3 Ov, Pt4 and Pt8 downregulate genes involved in stress pathways under control conditions. It could also show that Pt3 and Pt8 are more effective in lipid and specialized metabolism and Pt4 in folding, sorting and degradation, strategic processes in protein and *N*-glycan export pathways. However, at this first level of study, no correlation between a specific pathway and the morphotypes of *P. tricornutum* was observed.

**Fig. 1:**
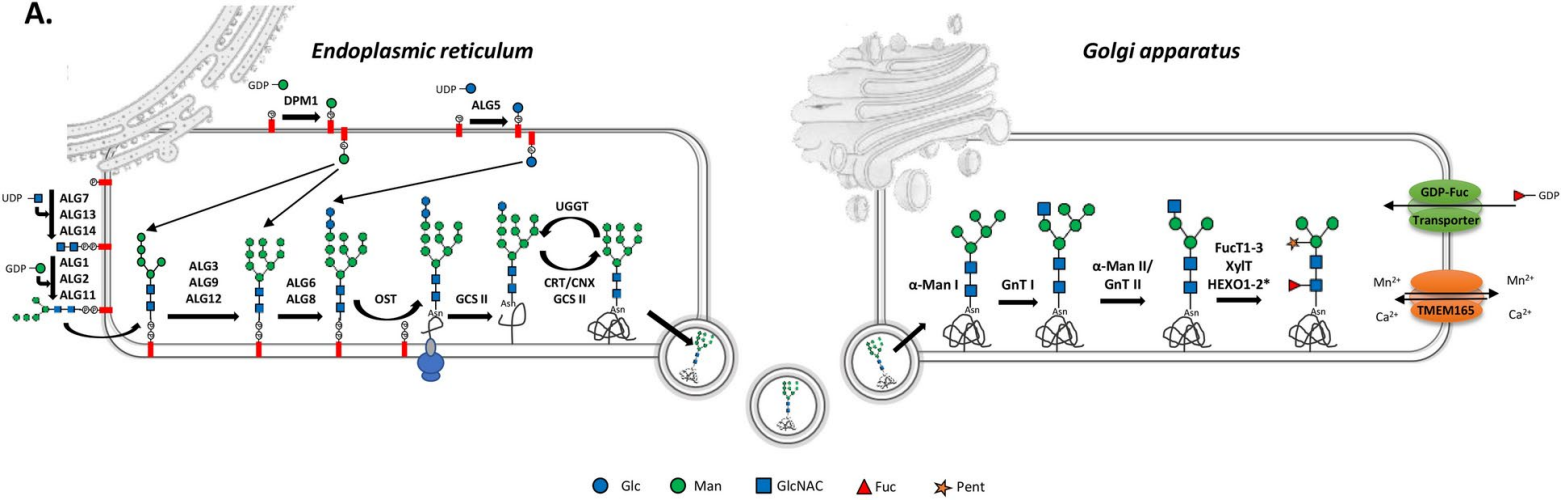

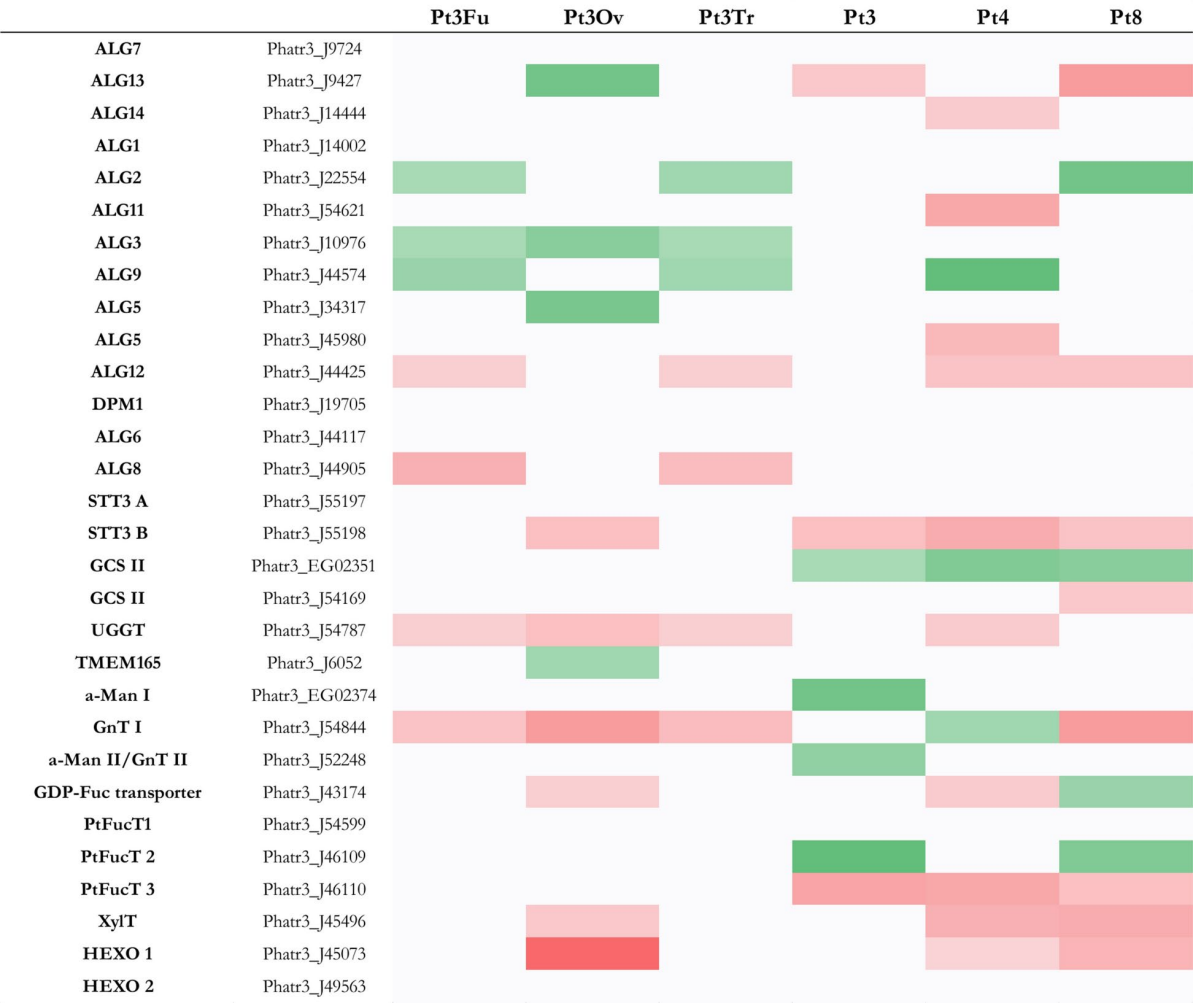
(**A**) Proposed *N*-glycan biosynthesis pathway in *P. tricornutum* and differential genes expression in the ten P. tricornutum strains. Representation of the putative mechanism of action of the different enzymes identified in the N-glycosylation biosynthesis pathway of *P. tricornutum*. The first steps take place in the endoplasmic reticulum leading to the synthesis of the lipid-linked oligosaccharide precursor, Glc2Man9GlcNAC2, which is then transferred en bloc onto the nascent protein thanks to the oligosaccharyltransferase. After the protein quality control steps, involving some chaperone proteins, the glycoprotein is transported to the Golgi apparatus where the N-glycan undergoes maturation steps leading to a paucimannosidic structure Pent1Fuc1Man3GlcNAC2. The N-glycan structures were drawn according to the Symbol Nomenclature for Glycans (Varki, 2017). Glc: glucose, Man: mannose, GlcNAC: N-acetylglucosamine, Fuc: fucose, Pent: Pentose like xylose or arabinose. HEXO1-2* : the localization of the hexosaminidases is not yet determined in P. tricornutum. (**B**). Genes implicated in the *N*-glycan biosynthesis pathway of *P. tricornutum*. In green, genes more expressed than the reference strain with significant fold change > 2 (i.e. log2FC > 1), pvalue < 0,05; in red genes less expressed than the reference strain with significant fold change < 0,5 (i.e. − 1 < log2FC), pvalue < 0,05; in white genes with non-significant fold change. Genes in Pt3 Fu, Pt3 Ov, Pt3 Tr, Pt3, Pt4 or Pt8 compared to Pt1.8.6.

**Fig. 2:**
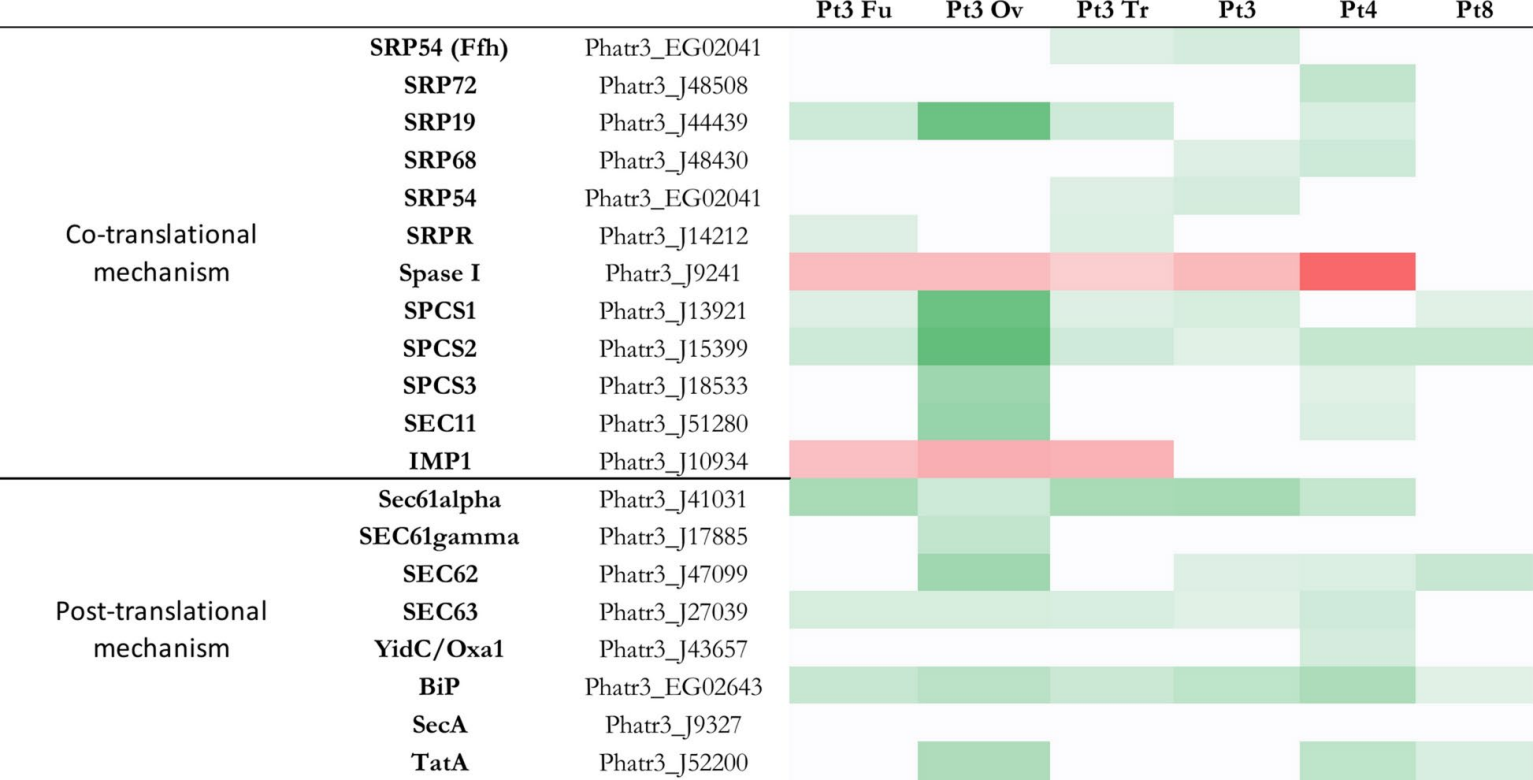
Heatmap of genes implicated in the protein export pathway of *P. tricornutum*. In green, genes more expressed than the reference strain with significant fold change > 2 (i.e. log2FC > 1), *p* value < 0,05; in red genes less expressed than the reference strain with significant fold change < 0,5 (i.e. − 1 < log2FC), *p* value < 0,05; in white genes with non-significant fold change. Genes in Pt3 Fu, Pt3 Ov, Pt3 Tr, Pt3, Pt4 or Pt8 compared to Pt1.8.6.

**Fig. 3:**
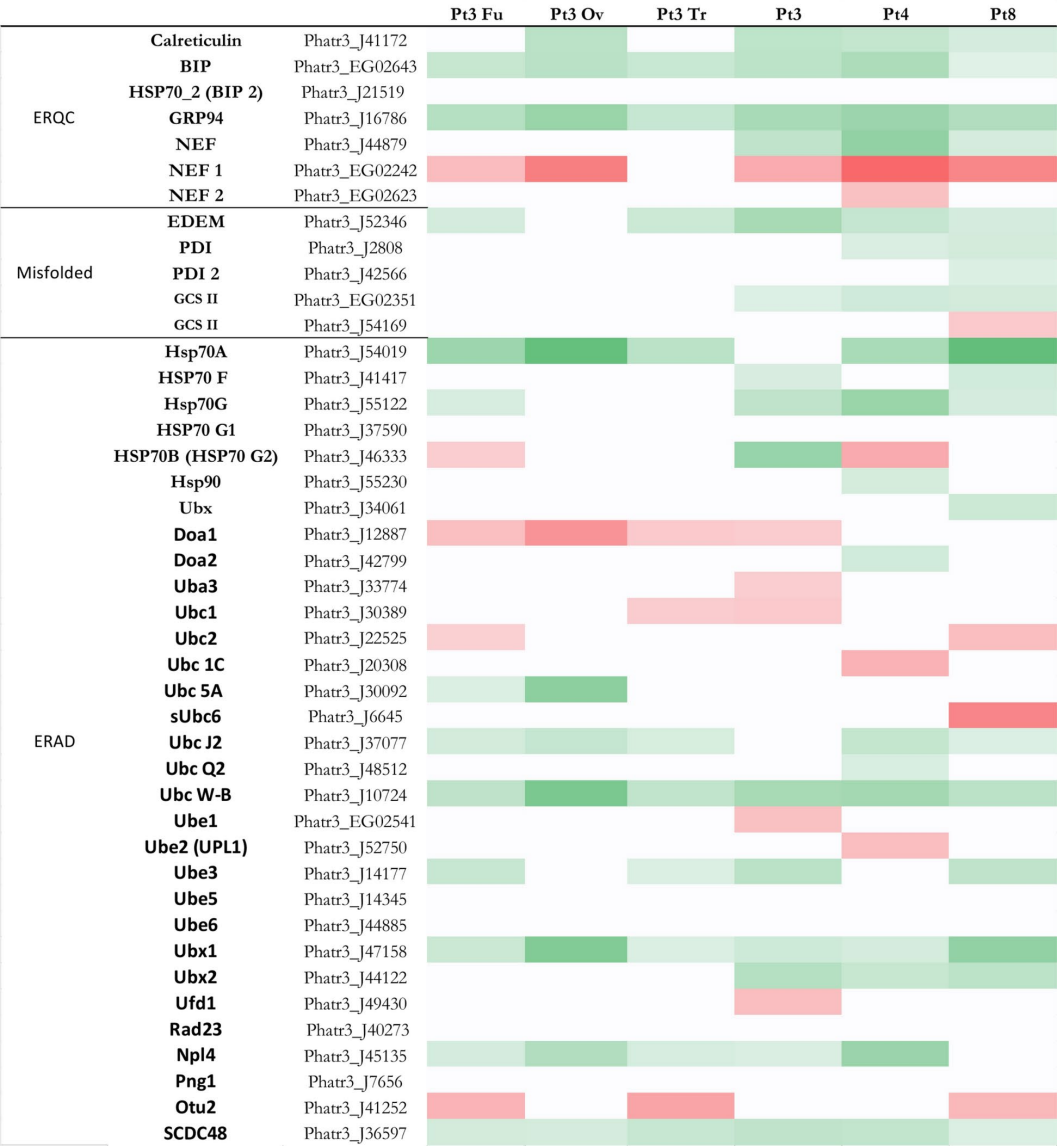
Heatmap of genes implicated in quality control of *P. tricornutum*. In green, genes more expressed than the reference strain with significant fold change > 2 (i.e. log2FC > 1), *p* value < 0,05; in red genes less expressed than the reference strain with significant fold change < 0,5 (i.e. − 1 < log2FC), *p* value < 0,05; in white genes with non-significant fold change. Genes in Pt3 Fu, Pt3 Ov, Pt3 Tr, Pt3, Pt4 or Pt8 compared to Pt1.8.6.

**Fig. 4:**
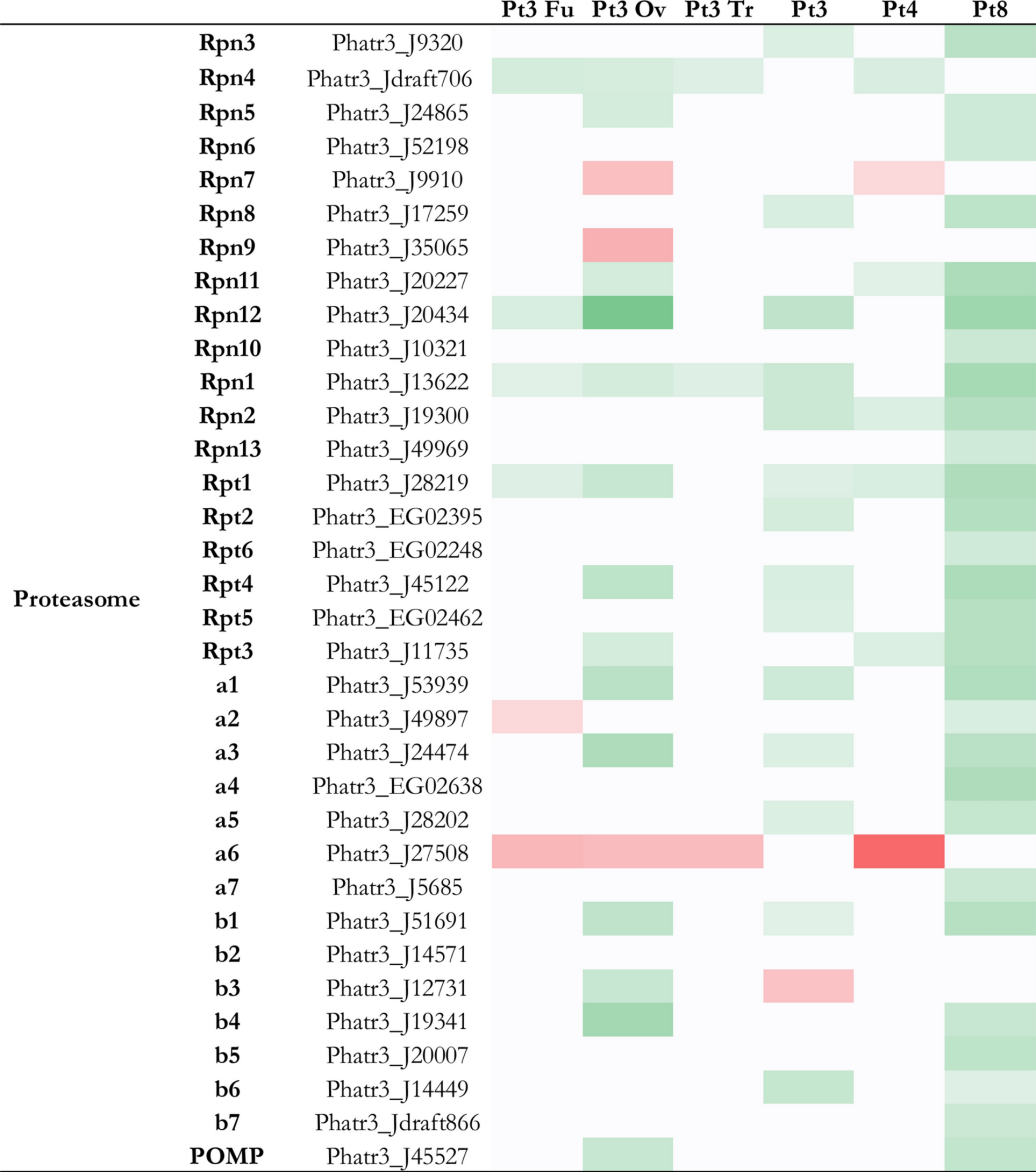
Heatmap of genes implicated in proteasome of *P. tricornutum*. In green, genes more expressed than the reference strain with significant fold change > 2 (i.e. log2FC > 1), *p* value < 0,05; in red genes less expressed than the reference strain with significant fold change < 0,5 (i.e. − 1 < log2FC), *p* value < 0,05; in white genes with non-significant fold change. Genes in Pt3 Fu, Pt3 Ov, Pt3 Tr, Pt3, Pt4 or Pt8 compared to Pt1.8.6.

To confirm the trend observed at this first level of investigation, we focused specifically on biological processes known in other organisms to have a significant impact on the production of Biologics and that can influence their critical quality attributes^46,47^. Therefore, we focused on the differential expression of genes involved in the *N*-glycan biosynthesis (Fig. 1), protein export and secretion (Fig. 2), protein quality control (Fig. 3), and proteasome (Fig. 4) in the different ecotypes of *P. tricornutum*.

### *N*-glycan biosynthesis

Most of the Biologics are glycoproteins for which the *N*-glycosylation is well known to influence their biological activities, potential immunogenicity and allergenicity^46,48^. Therefore, when selecting an organism as a host for the production of glycoproteins as Biologics, particular attention should be paid to the *N*-glycosylation pathway. The diatom *P. tricornutum* has been shown to be able to produce glycoproteins such as mAbs without immunogenic glycoepitopes on their *N*-glycans^10^. Moreover, these recombinant mAbs expressed in *P. tricornutum* have been shown to be glycosylated with mammalian-like oligomannosides resulting from processing steps in the endoplasmic reticulum (ER) and early Golgi apparatus (Fig. 1A). It is noteworthy that, despite the presence of glycoenzymes potentially involved in complex-type *N*-glycan biosynthesis within the Golgi apparatus of *P. tricornutum* (Fig. 1A), including *N*-acetylglucosaminyltransferase I (GnT I), fucosyltransferases or xylosyltransferases, a glycoproteomic study of the mAbs produced in *P. tricornutum* revealed that oligomannosidic *N*-glycan structures constitute the major *N*-glycan population of mAbs produced in *P. tricornutum*^10^. Based on this knowledge, we screened all the genes known so far to be involved in the different steps of the *N*-glycosylation pathway in *P. tricornutum* (Fig. 1B). Pt3 Ov, Pt4 and Pt8 have more differentially expressed genes (10, 13 and 12, respectively) involved in the *N*-glycosylation pathway as compared to the others, which present 7 differentially expressed genes. These are also the strains with the most under-expressed genes, with 6, 10 and 8 under-expressed genes, respectively, compared to the reference strain, Pt1.8.6 (Fig. 1B).

*N*-glycan synthesis is initiated in the ER with the synthesis of a dolichol-linked intermediate oligosaccharide precursor, commonly known as the lipid-linked oligosaccharide (LLO). The enzymes that synthesize this precursor, called ALGs (Asparagine-Linked Glycosylation), use nucleotide sugars and specific sugar acceptors to elongate the LLO (Fig. 1A). Among the 13 genes involved in the synthesis of this LLO, three are more highly expressed in the Pt3 Fu, Pt3 Ov and Pt3 Tr strains. The Pt3 Fu and Pt3 Tr strains have the same three genes that are more expressed than the reference, and the same two genes that are less expressed than the reference. The Pt4 and Pt8 strains have four and two genes, respectively, that are less expressed than in the reference strain, (Fig. 1B).

The Glc_2_Man_9_GlcNAc_2_-P-P-Dol precursor synthesized in *P. tricornutum* is then transferred *en bloc* to the asparagine at the consensus *N*-glycosylation sites (Asn-X-Thr/Ser/Cys), by an enzymatic complex called oligosaccharyltransferase (OST). This step takes place in the lumen of the ER. Two genes encoding putative catalytic subunits of the OST, named Phatr3_J55197 (SST3A) and Phatr3_J55198 (SST3B), have been identified in the *P. tricornutum’s* genome. The gene encoding SST3A is not differentially expressed between the different strains compared to the reference strain. In contrast, SST3B is less expressed in most samples, including Pt3 Ov, Pt3, Pt4 and Pt8. After being transferred by the OST, the *N*-glycan attached to the glycoprotein is then matured by two enzymes located in the lumen of the ER: the Glucosidase II (GCS II) and the UDP-glucose: glycoprotein glycosyltransferase (UGGT). Two predicted genes have been described to encode such GCS II in *P. tricornutum*: Phatr3_EG02351 gene, that is more expressed than the reference strain in Pt3, Pt4 and Pt8 and Phatr3_J54169, that is less expressed than the reference strain in Pt8 (Fig. 1B). The UGGT gene in *P. tricornutum* itself is less expressed in all the samples except in Pt3 and Pt8. Once correctly folded, the glycoproteins leave the ER to reach the Golgi apparatus, where maturation steps involve a variety of enzymes that result in species-specific *N*-glycan structures.

Upon entry into the Golgi apparatus, mannose residues are trimmed from the *N*-glycans by the alpha-mannosidase I, resulting in the Man_5_GlcNAc_2_ structure (Fig. 1A), which is the substrate for the GnT I. GnT I has been shown to be a key enzyme in the synthesis of complex *N*-glycan structures^49,50^. The gene encoding GnT I (Phatr3_J54844) is more expressed in the Pt4 strain than in the reference strain whereas it is less expressed in Pt3 Fu, Pt3 Ov, Pt3 Tr and Pt8 than in the reference strain (Fig. 1B). Therefore, the Pt4 strain might be a better choice for the production of recombinant glycoproteins carrying complex-type *N*-glycans as compared to others. However, the biochemical characterization of the anti-VHB mAbs produced in the Pt4 strain of *P. tricornutum* showed that mainly Man_9_GlcNAc_2_ and Man_8_GlcNAC_2_ *N*-glycans were found on the surface of the mAbs without any detection of complex-type *N*-glycans^10^. This suggests that there is no direct relationship between the expression level of GnT Iand the final N-glycan structure and that the maturation of the oligomannosidic *N*-glycans in the Golgi apparatus seems to be limited, at least on mAbs. Indeed, the absence of complex-type* N*-glycans on the surface of the mAbs could be explained by the nature of the protein, since the *N*-glycosylation sites of mAbs are buried within the CH_2_ constant domain. Thus, the folding of the mAbs limits the accessibility of the *N-*glycans to the Golgi processing glycosyltransferases, leading to incomplete maturation of the *N*-glycans. Nevertheless, the *N*-glycosylation profile of a total protein extract from the Pt1 strain showed that more mature *N*-glycans can be found. However, the complex *N*-glycans were present at low levelson the surface of *P. tricornutum* endogenous proteins^51^. Thus, it would be interesting in future studies to analyze the *N*-glycan profiles of all the ecotypes of *P. tricornutum*.

Special attention was also paid to fucosylation, as it has been previously shown that the α(1,3)-fucose glycoepitopes of plant-made biopharmaceuticals can lead to an immunogenic response in humans^52^. Looking at the fucosylation in *P. tricornutum*, it was demonstrated that the overexpression of PtFucT1 leads to a significant increase in α(1,3)-fucosylation on *P. tricornutum* endogenous glycoproteins^53^. In the present work, the expression of the gene encoding PtFucT1 (Phatr3_J54599) is not modified within the different ecotypes. In contrast, the putative PtFucT2 is more expressed in Pt3 and Pt8 than in the reference strain while the expression of the gene encoding for the putative PtFucT3 is less expressed in Pt3, Pt4, and Pt8 than in the reference strain. The GDP-Fuc transporter of *P. tricornutum* has also been characterized and shown to restore fucosylation of proteins in a mutant CHO cell line lacking its endogenous GDP-Fuc transporter activity^53^. The gene encoding for this transporter (Phatr3_J43174) is less expressed in Pt3 Ov and Pt4 than in the reference strain, but more expressed in Pt8 than in the reference strain (Fig. 1B). Once again, the Pt4 strain seems to be more interesting for the production of Biologics dedicated to human health, since the genes involved in the addition of immunogenic epitopes are less expressed in this ecotype.

The regulation of the processing of oligomannosidic to complex type *N-*glycans depends on the accessibility of the *N*-glycans to glycosyltransferases (GTs) and glycosidases in the Golgi apparatus, but also on the regulation of these enzymes by the Golgi Mn^2+^ homeostasis^54^. Recent studies carried out on human congenital disorders of glycosylation have highlighted the role of TMEM165 (transmembrane protein 165) in Golgi cation homeostasis and the impact of *TME165* deficiency on the *N*-glycan processing^55,56^. *TMEM165* homologous sequences have been identified in several eukaryotic organisms including *P. tricornutum* but their role is not yet understood^54^. The gene Phatr3_J6058 from *P. tricornutum* encodes a putative homolog of TMEM165, which is only more expressed in Pt3 Ov than in the reference strain compared to other strains or morphotypes (Fig. 1B). The presence of TMEM165 in organisms that do not produce many complex glycans, such as *Phaeodactylum,* and its overexpression in stress-related morphotypes could potentially suggest a role in osmoregulation. Indeed, comparative EST-based analyses showed that the oval morphotype exhibited an up-regulation of genes encoding proteins involved in stress responses with putative roles in signaling, membrane remodeling, protein degradation but also in cell homeostasis^38^. More recently, Kleiner et al*.,* have also shown an increase in cytosolic Ca^2+^ concentration in *P. tricornutum* in response to cold stress^57^.

### Protein export and secretion

In mammalian cell factories, it is well known that improving the efficiency and stability of recombinant protein production relies on maximizing the protein export and secretory capacity of the cells^47^. Recently, it has been shown that microalgae are also capable of secreting recombinant proteins into their culture media^9,58,59^, including *P. tricornutum*^5,7^. The protein export is the active transport of proteins from the cytoplasm to the outside of the cell. Both prokaryotic and eukaryotic organisms have two major modes of protein export: (1) the co-translational delivery mechanism, in which the nascent polypeptide can be delivered to the export membrane along with the ribosome during protein synthesis, or (2) the post-translational mechanism, in which protein delivery can occur after its synthesis is complete^60^. In *P. tricornutum*, the predicted genes related to protein export and secretion that have been described are derived from both prokaryotes and eukaryotes. In all strains, most of the genes involved in the co- and post-translational delivery mechanism are more expressed than the reference strain, especially in Pt3 Ov and Pt4, which have the highest number of genes more expressed than the reference strain compared to other strains, with 11 and 12 genes overexpressed, respectively (Fig. 2).

In the co-translational mechanism, the peptide chains are first synthesized by the ribosome. Then, signal recognition particles (SRPs) recognize and bind to the signal peptide. Simultaneously with the signal peptide binding, the SRP recognizes the SRP receptor (SRPR) in the ER membrane and then forms a complex with the ribosome on the membrane. Among the SRPs, SRP19 is the most differentially expressed among the six different accessions, especially in Pt3 Ov. Only Pt3 Fu and Pt3 Tr present a significant differential expression for SRPR, which it is more expressed than the reference strain in both strains (Fig. 2). Pt8 has no gene related to SRP or SRPR that is differentially expressed. Subsequently, the translocation of the peptide occurs, leading to the entry of the peptide into the lumen of the ER. During this process, signal peptides are removed by the signal peptidase in the lumen of the ER. In prokaryotes, signal peptidases (SPase I and II) are involved. In eukaryotes, this step is performed by signal peptidase complex subunits (SPCS 1, 2 and 3), signal peptidase I (SEC11) and mitochondrial inner membrane protease subunits (IMP)^60^. Our study reveals that *Spase I* is less expressed than the reference strain in all strains except for Pt8, where no significant differential expression was observed. *IMP1* is also less expressed than the reference strain in Pt3 Fu, Pt3 Ov and Pt3 Tr, no significant differential expression was observed in the other strains compared to the reference strain. Moreover, in Pt3 Ov, all the other genes (*SPC1, SPC2, SPC3* and *SEC11*) are more expressed than the reference strain, in the other strains only two or three of them are more expressed than the reference strain. *SPCS2* is more expressed than the reference strain in all strains.

Regarding the genes involved in the post-translational mechanism, they are all more expressed than the reference strain or with no significant difference in all strains. Moreover, Pt3 Ov and Pt4 are the most predominant with six out of eight genes (Fig. 3). In eukaryotic organisms, the post-translational mechanism involves the translocation channel consisting of SEC61, SEC62, SEC63 (transport protein Sec61 subunits alpha/gamma and translocation proteins) and BIP (an ER chaperone). All these actors are more expressed in Pt3 Ov compared to the reference strain, and BIP is more expressed in all the strains compared to Pt1.8.6. In prokaryotic organisms, this mechanism involves, among others, the membrane protein insertase of the YidC/Oxa1 family and the preprotein translocase subunit SecA^60^. Almost no difference in expression is observed for these two actors. Finally, another protein transport system that transports folded proteins in bacteria, archaea, and chloroplasts is the twin-arginine translocation (Tat) pathway, which involves several membrane proteins with overlapping functions, of which TatA is the most important^61^. In our study, Tat A was more expressed than the reference strain only in Pt3 Ov, Pt4 and Pt8, while the other three strains did not show any significant expression.

The fact that more genes involved in the protein export pathway are more expressed in Pt3 Ov seems to correlate with previous results. Indeed, it has been shown that more proteins are expressed at higher levels in oval cell cultures compared to triradiate or fusiform cell cultures^32,41^. A proteomic analysis of the secretome of the three morphotypes of Pt3 reveals that 385 proteins are secreted in the culture media of Pt3 Ov, compared to 275 and 231 proteins secreted for Pt3 Fu and Pt3 Tr, respectively^32^. A study led by Galas et al., in 2021 also confirmed that Pt3 Ov cells can secrete proteins faster and in higher amounts than the other two morphotypes^40^. As mentioned above, since secretory capacity is important for the Biologics accumulation in the culture medium, it seemed important to focus on protein secretion, regulation of exocytosis and vesicle docking involved in exocytosis. However, very little data are currently available on identified or predicted genes involved in these pathways in *P. tricornutum*. Since this microalgae is a metabolic intermediate between plants and mammals^1^ and to extend our knowledge of the secretion and exocytosis process in *P. tricornutum*, we performed a BLAST analysis on the *Phaeodactylum* genome to identify homologous genes compared to *A. thaliana* (AT) and *Homo sapiens* (HS) genes that are involved in these pathways. The expression of the identified genes was then described in the selected RNA-Seq (Supplemental 2 and 3). It was noted that Pt4 has more genes involved in ‘protein secretion and exocytosis’ that are more expressed than the reference strain (8 genes). Pt3 Ov has specifically more expressed genes than the reference strain, such as Ras-related protein RAB8 (Phatr3_J22713) and Kish-A-like protein (Phatr3_J12535). Pt4 has specifically more expressed than the reference strain genes such as Ras-related protein RABE1e (Phatr3_J22095), Golgi apparatus membrane protein-like ECHIDNA (Phatr3_J6254), PHF1 (a SEC12-like protein 1) (Phatr3_J7966), and exocyst complex component SEC3A (Phatr3_EG02390). Finally, three Ras-related proteins, Rab-10 (Phatr3_J44216), Rab-8A (Phatr3_EG02235) and Rab-3C (Phatr3_J22095) are specifically more expressed in Pt4 than in the reference strain.

Regarding the expression of genes involved in the protein export pathway and the secretion, Pt3 Ov and Pt4 seem to have more potential to enhance the protein production and its export in the culture media.

### Quality control and proteasome

Before leaving the ER and being delivered to various intracellular compartments or the extracellular environment, proteins interact with several quality control factors whose aim is to release well-folded, functional proteins. Analysis of the DEGs encoding these factors reveals that Pt3, Pt4, and Pt8 have more more-expressed genes than the reference strain that are involved in the protein quality control than others (15, 18, and 18 genes respectively) (Fig. 3). Among these factors, nascent proteins interact with chaperones such as calreticulin, calnexin, heat shock proteins (HSP) such as BiP (Binding Immunoglobulin Protein) and GRP (Glucose related proteins). The genes encoding BiP (Phatr3_EG02643) and GRP94 (Phatr3_J16786) are more expressed in all strains compared to Pt1.8.6, but there is no significant differential expression of the gene encoding HSP70_2 (Bip2–Phatr3_J21519). Recently, some studies have demonstrated a correlation between BiP expression levels and mAb production in mammalian cell lines producing mAbs^62,63^. For *N*-glycosylated proteins, the oligosaccharide on their surface is recognized by two other ER luminal enzymes: glucosidases I and II (GCSI and GCSII)^64^. These enzymes cleave the terminal glucose residues. The GCSI cleaves the first terminal residue and the GCSII cleaves the other two. Before the last glucose residue is trimmed, the glycoprotein is recognized by the chaperone proteins and, if properly folded, the third glucose is removed by the GCSII and the protein is directed to the secretory pathway. The gene Phatr3_EG02531, encoding a GCSII is more highly expressed in Pt3, Pt4 and Pt8 than in the reference strain. Misfolded proteins whose three glucoses are cleaved are recognized by UGGT (UDP-glucose/glycoprotein glucosyltransferase), which adds glucose residues to these misfolded proteins to send them back to the chaperones. If the protein is still misfolded after the ER quality control cycle, it is recognized by α1,2-mannosidase I via the EDEM (ER degradation enhancing 1,2-mannosidase protein).The gene Phatr3_J52346, which encodes the EDEM protein, is expressed at higher levels than Pt1.8.6 in all strains except Pt3 Ov, for which the expression is not significantly different from Pt1.8.6. The α1,2-mannosidase I specifically cleaves mannose residues, allowing the misfolded proteins to be directed to the ER-associated degradation (ERAD)^64^. It has been shown that, among other stress factor, the expression of proteins known to be difficult to produce, such as mAbs, can disrupt the ER homeostasis, leading to the accumulation of misfolded proteins, and subsequently of the activation of the ERAD pathway to restore the ER homeostasis^62,63^. Regarding the ERAD pathway, Pt4 has more genes more expressed than the reference strain (8 genes) and Pt3 has more l genes less expressed than the reference strain. The genes encoding Ubc W-B (Phatr3_J10724), Ubx1 (Phatr3_J47158) and SCDC48 (Phatr3_J36597) are more expressed in the seven strains than in the reference strain.

In the ERAD pathway, most substrate proteins are ubiquitin-modified prior to proteasomal degradation. The proteasome is a protein-degrading apparatus composed of two subcomplexes: a 20S core particle (CP), which carries the catalytic activity and a regulatory 19S regulatory particle (RP). The CP is composed of heptameric rings: outer identical α-rings (a1 to 7) and inner identical β-rings (b1 to 7) and a maturation protein (POMP). The RP consists of two eight-subunit subcomplexes: the lid (Rpn3 to Rpn12), and the base (Rpn1, 2 and 13, Rpt 1 to 6). Rpn10 can interact with either the lid or the base, stabilizing the interaction between the two^65^ Regarding the proteasome, Pt8 has more and only more expressed genes than the reference strain compared to others (i.e. 28 genes) (Fig. 4), followed by Pt3 Ov and Pt3, which both have 14 genes that are more expressed compared to Pt1.8.6. A correlation between the overexpression of QC-related genes, especially those involved in ERAD, and the overexpression of proteasome-related genes could be expected, since the activation of the proteasome follows that of the ERAD pathway. This correlation is observed for Pt8, which has the most overexpressed genes in both cases. However, this is not the case for Pt3 Ov and Pt3.

Considering the elements provided by the analysis of DEGs involved in QC and proteasome, we can suggest that the Pt3 Fu and Pt3 Tr could be a bit more interesting for the production of Biologics. Indeed, these strains have more down-regulated genes and less up-regulated genes that are involved in QC and proteasome than the other strains. It can be assumed that the lower activation of genes related to these pathways implies a better capacity to produce correctly folded proteins.

### Proteases

As mentioned above, the mAbs production yields obtained in *P. tricornutum* are approximately 40-fold lower than the 10 g/L observed in CHO cells^18^. The currently observed low yields may be related to the proteolytic degradation of mAbs accumulated in the culture medium. Although host cell proteases are critical due to their essential functions in catalytic and metabolic pathways, as well as in the renewal of extracellular waste^66^, when present in the cell culture medium they can degrade the product of interest^67^. Indeed, previous studies in plants or CHO have mentioned that proteolytic degradation of mAbs that accumulate in the culture media of cell expression systems can dramatically affect the yield of some recombinant proteins^67,68^. Proteases can accumulate in the culture media either by secretion from the host cell or by release during cell lysis. Moreover, an in silico gene analysis^69^ and a proteomic analysis^32^ of the secretome of *P. tricornutum* suggest that this microalga secretes a large number of putative proteases. It is essential to consider the occurrence of proteases in the selected datasets (Supplemental 4).

**Fig. 5:**
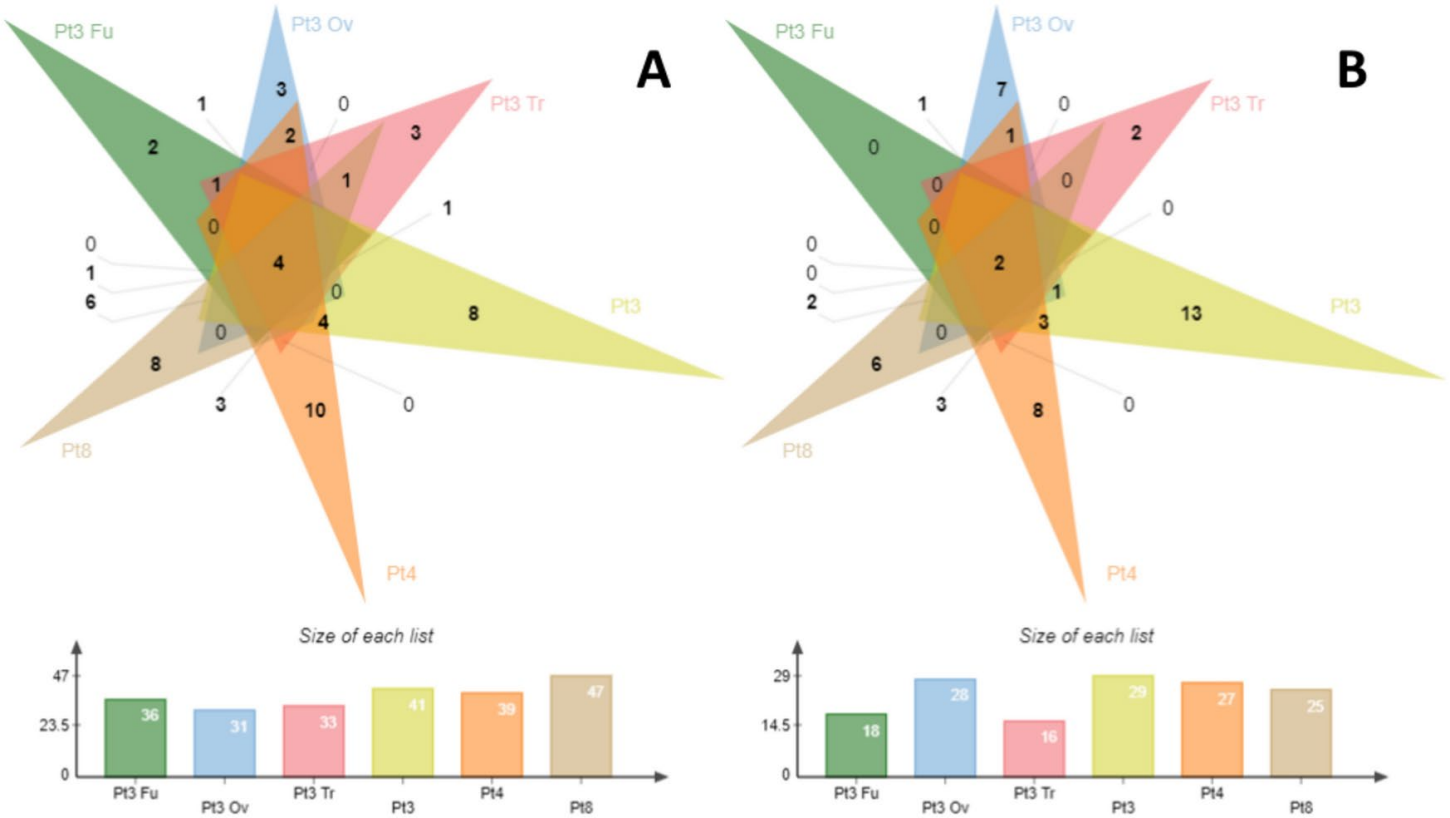
Venn diagram of genes implicated in protease activities in *P. tricornutum*. (**A**) More expressed genes than the reference strain in Pt3 Fu, Pt3 Ov, Pt3 Tr, Pt3, Pt4 or Pt8 compared to Pt1.8.6. (**B**) Less expressed genes than the reference strain in Pt3 Fu, Pt3 Ov, Pt3 Tr, Pt3, Pt4 or Pt8 compared to Pt1.8.6.

First, for all strains, most of the identified proteases have no significant expression (about 110 out of the 173 identified proteases). Then, the overall number of proteases is relatively well balanced between the ecotypes for more expressed than the reference strain genes (about 35 proteases) or less expressed than the reference strain genes (about 25) (Supplemental 4). We then wanted to know if there were any proteases that were specifically expressed in each strain. For the proteases more expressed than the reference strain, 2, 3, 3, 8, 10, and 8 proteases were found only in Pt3 Fu, Pt3 Ov, Pt3 Tr, Pt3, Pt4, and, Pt8 respectively. For proteases less expressed than the reference strain, 7, 2, 13, 8 and 6 DEGs were found only in Pt3 Ov, Pt3 Tr, Pt3, Pt4, and, Pt8, respectively (Fig. 5). Finally, among these specific proteases, we identified (in red) those secreted according to Chuberre et al. 2021 and Dorrell et al. 2021 (Table 3). Our analysis showed that Pt3 and Pt4 have the most specifically secreted proteases, which are less expressed than the reference strain (six proteases). However, Pt3 is also the strain with the most specifically secreted proteases that are more expressed than the reference strain (five proteases), while Pt4 has only two. It is also worth mentioning that Pt3 Ov has 2 secreted proteases more expressed than the reference strain and 4 secreted proteases less expressed than the reference strain. The protease categories identified include serine-type proteases, cysteine-type proteases, metalloproteases, ATP-dependent proteases, and thiol-dependent proteases. In all strains, at least one gene encoding a secreted serine-type protease is specifically more expressed than in the reference strain. Serine-type proteases are commonly released as host cell proteins (HCPs) into the culture media, causing the degradation of recombinant proteins during production^70,71^. Their removal during purification is often challenging, with some, even co-eluting with mAbs during protein A chromatography^72^.

**Table 3:** Genes of proteases more or less expressed only found in Pt3 Fu, Pt3 Ov, Pt3 Tr, Pt3, Pt4 or Pt8 (compared to Pt1.8.6). In red, secreted proteases identified according to Chuberre et al., 2021 and Dorrell et al., 2021).

	Genes of proteases up-regulated		Genes of proteases down-regulated	
	Gene ID	Predicted protease	Gene ID	Predicted protease
Pt3 Fu	Phatr3_EG02027 Phatr3_J11204	Extracellular metalloprotease Serine-type carboxypeptidase	-	-
Pt3 Ov	Phatr3_J44664 Phatr3_J50206 Phatr3_J31772	Cysteine-type peptidase Serine-type endopeptidase Thiol-dependent deubiquitinase	Phatr3_J12595 Phatr3_J40690 Phatr3_J24006 Phatr3_J42588 Phatr3_J13331 Phatr3_J46562 Phatr3_J3924	Metalloaminopeptidase Metalloendopeptidase Metalloendopeptidase Metalloendopeptidase Serine-type peptidase Serine-type peptidase Thiol-dependent deubiquitinase
Pt3 Tr	Phatr3_J11217 Phatr3_J38448 Phatr3_J46548	ATP-dependent Clp protease Metalloaminopeptidase serine-type endopeptidase	Phatr3_J48319 Phatr3_J47594	Metalloprotease Serine-type endopeptidase
Pt3	Phatr3_J12595 Phatr3_EG02434 Phatr3_J12106 Phatr3_J12241 Phatr3_J5396 Phatr3_J35781 Phatr3_J3061 Phatr3_J42729	Metalloaminopeptidase Metalloaminopeptidase Metalloendopeptidase Peptidase Peptidase Protease Serine-type peptidase Ubiquitin carboxyl-terminal hydrolase	Phatr3_J9388 Phatr3_J16168 Phatr3_J11217 Phatr3_J25433 Phatr3_J4936 Phatr3_J44664 Phatr3_J42694 Phatr3_J49223 Phatr3_J14380 Phatr3_J49652 Phatr3_J44745 Phatr3_J31772 Phatr3_EG02322	Aspartic-type endopeptidase Aspartic-type endopeptidase Clp protease Cysteine-type peptidase Cysteine-type peptidase Cysteine-type peptidase Lys48-specific deubiquitinase Serine-type endopeptidase Serine-type endopeptidase Serine-type peptidase Thiol-dependent deubiquitinase Thiol-dependent deubiquitinase Thiol-dependent deubiquitinase
Pt4	Phatr3_J9625 Phatr3_J10593 Phatr3_J45606 Phatr3_J13602 Phatr3_EG02320 Phatr3_J46087 Phatr3_J46562 Phatr3_J46565 Phatr3_J29340 Phatr3_Jdraft1763	Aspartic-type endopeptidase ATP-dependent Clp protease Metalloendopeptidase Metallopeptidase Metallopeptidase Serine-type endopeptidase Serine-type peptidase Serine-type peptidase Thiol-dependent deubiquitinase Thiol-dependent ubiquitinyl hydrolase activity	Phatr3_J1884 Phatr3_J38448 Phatr3_J45960 Phatr3_J48491 Phatr3_J4014 Phatr3_J40462 Phatr3_J42439 Phatr3_J48493	Metalloaminopeptidase Metalloaminopeptidase Serine-type carboxypeptidase Serine-type endopeptidase Serine-type endopeptidase Serine-type endopeptidase Serine-type endopeptidase Serine-type endopeptidase
Pt8	Phatr3_J16455 Phatr3_EG02217 Phatr3_J9309 Phatr3_J8670 Phatr3_J45960 Phatr3_J47107 Phatr3_J13240 Phatr3_J4014	clpC peptidase Cysteine-type peptidase Metalloendopeptidase Metalloendopeptidase Serine-type carboxypeptidase Serine-type endopeptidase Serine-type endopeptidase Serine-type endopeptidase	Phatr3_J9625 Phatr3_J45606 Phatr3_J42587 Phatr3_J50206 Phatr3_J50346 Phatr3_J37460	Aspartic-type endopeptidase Metalloendopeptidase Metalloendopeptidase Serine-type endopeptidase Serine-type endopeptidase Serine-type peptidase

Considering the level of information provided by the expression of proteases, and especially of those secreted in the culture media, the Pt4 ecotype seems to be more interesting for the production of Biologics, given the low number of less expressed than the reference secreted proteases and the high number of more expressed than the reference secreted proteases. Thus, this accession is likely to lead to a higher accumulation of recombinant proteins in the culture medium. Nevertheless, it would be interesting to precisely identify the proteases directly involved in the degradation of recombinant proteins, in order to strengthen the conclusion regarding the interest of one strain over another.

## Conclusion

In conclusion, our meta-analysis of publicly available transcriptomic data from different strains of *P. tricornutum* showed that compared to the others, Pt3 Ov, Pt4 and Pt8 seemed to be more efficient in the *N*-glycosylation pathway, Pt3 Ov and Pt4 in protein export and secretion, Pt3 Tr and Pt3 Fu in quality control and proteasome and Pt3 Ov and Pt4 were less affected by secreted proteases. Therefore, based on this analysis and considered it as a whole, Pt4 appears to be the optimal strain for the production of recombinant proteins, confirming existing data that identify the Pt4 strain as the most promising for the production of Biologics^5–7,41,42,73^, followed by Pt3 Ov^32,40^. It is worth mentioning that the Pt8 strain, which also has interesting characteristics even though, has never been mentioned for the production Biologics. In addition, it is important to note that the laboratory conditions to which the strains are exposed, may favor mutations, especially due to a high growth rate, which has already been observed in strains coming from different laboratories^74^. For example, it has recently been shown that Pt1 strains issued from two different laboratories, present different lipid contents^42^. This is certainly a limitation of this meta-analysis, which uses datasets from different RNA-seq studies with similar but not strictly identical culture conditions. Due to our selection criteria for RNA-seq data regarding the closest similarity of culture conditions, this meta-analysis concerns only 4 different *P. tricornutum* ecotypes, whereas 10 ecotypes are currently commonly described in the literature. Recently, an RNA-seq analysis of the 10 most commonly used *P. tricornutum* strains cultured under exactly the same conditions was published^75^. This study in agreement with our meta-analysis, demonstrated that the Pt4 strain appears to be the most promising for Biologics production^75^. In future work, it would be interesting to produce antibodies in the different ecotypes of *P. tricornutum* and evaluate whether these strains are still the most interesting on an industrial scale. Thus, further work needs to be performed in the coming years to understand and better control the cell biology of *P. tricornutum* in order to finally improve the Biologics production yield in this diatom.

Methods.

### Identification and selection of the RNA-seq datasets

RNA-seq studies used were chosen according to (1) procedures of culture, (2) ecotypes and (3) morphotypes. The chosen culture conditions were those available where microalgae cells were cultured in natural or artificial seawater media supplemented with Conway or f/2, at a temperature comprised between 18 and 20 °C and a photoperiod of 12:12 or 16:8 with light intensity comprised between 40 and 175 μmol m^−2^ s^−1^ and harvested on the 6th to 8th day of culture. The chosen datasets are from the three morphotypes (oval, fusiform and triradiate as well as enriched versions of these morphotypes) (Table 1). The datasets used are those of cells grown without any stress (control). All libraries used were sequenced on an Illumina instrument. Based on those search criteria, seven RNA-seq datasets were included in our study. All information about these datasets is summarized in Table 1. FASTQ files were downloaded from the Sequence Read Archive (SRA)-NCBI (https://www.ncbi.nlm.nih.gov/sra/docs/sradownload). A PRISMA checklist and flow diagram are available in Supplemental data S5.

### Trimming, alignment, and gene count

FastQ files were uploaded on the Galaxy platform^76^ (https://plants.usegalaxy.eu), and trimmed with Trimmomatic V0.36.6. Reads were aligned to the *P. tricornutum* genome (Phaeodactylum_tricornutum.ASM15095v2) with Hisat2 V2.1.0 and a gene counting was done with feature-counts V1.6.4. The alignment results showed that 64.3–80.1% of the reads were assigned to unique positions on the reference genome (Supplemental data S6).

### Differential gene expression analysis

Analyses of the differential gene expression (Pt3 Fu, Pt3 Ov, Pt3 Tr, Pt3, Pt4 or Pt8) were performed against Pt1.8.6 with DESeq2 V2.11.40.6 (which also normalized the datas) and visualized by volcano plots (DESeq2), heatmaps (Microsoft Excel) and Venn diagrams (JVenn https://jvenn.toulouse.inrae.fr/app/example.html). The chosen Fold Change (FC) was 0,5 < FC > 2 (i.e. − 1 < log2FC > 1) with a *p* value < 0,05 (i.e. log10 *p* value > 1,3).

### Gene ontology

Gene ontology was explored using the overrepresentation test performed through ShinyGO 0.76 (http://bioinformatics.sdstate.edu/go/, Pathway database: KEGG, FDR cutoff 0.05). Genes involved in protease activities were obtained from UniprotKB (keyword: KW-064 Protease) and from DiatomicBase (https://www.diatomicsbase.bio.ens.psl.eu/resources).

## Supplementary Information

Supplementary Information.

## Data Availability

All information on these datasets is summarized in Table 3 and a PRISMA checklist and flow diagram are available in Supplemental data S5. All data analysed during this study are included in these published articles: 10.1111/nph.13787 for Pt1.8.6, 10.1038/s41598-018-32519-7 for enriched Pt3, 10.1038/s41598-018-23106-x for natural Pt3, 10.1093/pcp/pcx127 for Pt4, and 10.1111/nph.1712 for Pt8.
