# Supplementary material for: Meta-analysis of RNA-Seq datasets allows a better understanding of P. tricornutum cellular biology, a requirement to improve the production of Biologics
Source: Sci Rep. 2025 Jan 29;15:3603. doi: 10.1038/s41598-025-87620-5 (PMC11775308; doi:10.1038/s41598-025-87620-5)
Supplement: Supplementary file 1 — Supplementary Information. [file 41598_2025_87620_MOESM1_ESM.docx]

**Supplemental data:**

**Supplemental data 1.** Differential expression analysis of genes in the different ecotypes of *P. tricornutum*. Volcanoplots obtained from transcriptomic data. Red points and numbers correspond to up regulated genes in Pt3 Fu, Pt3 Ov, Pt3 Tr, Pt3, Pt4 or Pt8 compared to Pt1.8.6 used as a reference. Blue points and numbers correspond to down-regulated genes in Pt3 Fu, Pt3 Ov, Pt3 Tr, Pt3, Pt4 or Pt8 compared to Pt1.8.6. Differentially expressed genes have a fold change ≤ 0.5 or fold change ≥ 2 (i.e -1 <log2FC> 1) and p-value ≤ 0.05 ((i.e log10pvalue> 1,3).

**Supplemental data 2.** Heatmap of genes implicated in protein secretion, regulation of exocytosis and vesicle docking involved in exocytosis of P. tricornutum after BLAST on genes of *A. thaliana* (AT) identified according to TAIR. In green, genes up-regulated with significant fold change > 2 (i.e log2FC> 1), p-value<0,05; in red genes down-regulated with significant fold change <0,5 (i.e -1 <log2FC), p-value<0,05; in white genes with non-significant fold change. Genes in Pt3 Fu, Pt3 Ov, Pt3 Tr, Pt3, Pt4 or Pt8 compared to Pt1.8.6.

**Supplemental data 3.** Heatmap of genes implicated in protein secretion, regulation of exocytosis and vesicle docking involved in exocytosis of P. tricornutum after BLAST on genes of Homo sapiens (HS) identified according to UNIPROT KB. In green, genes up-regulated with significant fold change > 2 (i.e log2FC> 1), p-value<0,05; in red genes down-regulated with significant fold change <0,5 (i.e -1 <log2FC), p-value<0,05; in white genes with non-significant fold change. Genes in Pt3 Fu, Pt3 Ov, Pt3 Tr, Pt3, Pt4 or Pt8 compared to Pt1.8.6.

**Supplemental data 4.** Number of genes implicated in protease activities in P. tricornutum. Genes involded in protease activities were obtained from UniprotKB (keyword : KW-064 Protease) and from DiatomicBase. Genes in Pt3 Fu, Pt3 Ov, Pt3 Tr, Pt3, Pt4 or Pt8 compared to Pt1.8.6.

**Supplemental data 5.** PRISMA checklist, abstract checklist and flow diagram

**Supplemental data 6.** Alignment results of the different sequencing on reference genome (Phaeodactylum_tricornutum.ASM15095v2) with Hisat2 V2.1.0.
